# Therapeutic targeting of Notch signaling and immune checkpoint blockade in a spontaneous, genetically heterogeneous mouse model of T-cell acute lymphoblastic leukemia

**DOI:** 10.1242/dmm.040931

**Published:** 2019-09-01

**Authors:** Jie Gao, Michael Van Meter, Susana Hernandez Lopez, Guoying Chen, Ying Huang, Shumei Ren, Qi Zhao, Jose Rojas, Cagan Gurer, Gavin Thurston, Frank Kuhnert

**Affiliations:** Regeneron Pharmaceuticals, Inc., Tarrytown, New York, NY 10591, USA

**Keywords:** T-ALL, Notch1, Targeted therapy, PD-1 blockade, Immunotherapy

## Abstract

T-cell acute lymphoblastic leukemia (T-ALL) is an aggressive hematologic cancer derived from the malignant transformation of T-cell progenitors. Outcomes remain poor for T-ALL patients who have either primary resistance to standard-of-care chemotherapy or disease relapse. Notably, there are currently no targeted therapies available in T-ALL. This lack of next-generation therapies highlights the need for relevant preclinical disease modeling to identify and validate new targets and treatment approaches. Here, we adapted a spontaneously arising, genetically heterogeneous, thymic transplantation-based murine model of T-ALL, recapitulating key histopathological and genetic features of the human disease, to the preclinical testing of targeted and immune-directed therapies. Genetic engineering of the murine *Notch1* locus aligned the spectrum of *Notch1* mutations in the mouse model to that of human T-ALL and confirmed aberrant, recombination-activating gene (RAG)-mediated 5′ *Notch1* recombination events as the preferred pathway in murine T-ALL development. Testing of Notch1-targeting therapeutic antibodies demonstrated T-ALL sensitivity to different classes of Notch1 blockers based on Notch1 mutational status. In contrast, genetic ablation of Notch3 did not impact T-ALL development. The T-ALL model was further applied to the testing of immunotherapeutic agents in fully immunocompetent, syngeneic mice. In line with recent clinical experience in T-cell malignancies, programmed cell death 1 (PD-1) blockade alone lacked anti-tumor activity against murine T-ALL tumors. Overall, the unique features of the spontaneous T-ALL model coupled with genetic manipulations and the application to therapeutic testing in immunocompetent backgrounds will be of great utility for the preclinical evaluation of novel therapies against T-ALL.

## Abstract (precis)

**Summary:** Adapting a spontaneous, genetically heterogenous T-ALL model to preclinical testing demonstrated that response to therapeutic anti-Notch1 antibodies was determined by Notch1 mutational status and that PD-1 immune checkpoint blockade alone lacked anti-tumor activity.

## INTRODUCTION

T-cell acute lymphoblastic leukemia (T-ALL) is an aggressive hematologic tumor which arises from the malignant transformation of T-cell progenitors (Belver and Ferrando, 2016; Vadillo et al., 2018). The disease accounts for 15-25% of acute lymphoblastic leukemia cases and affects both children and adults. The implementation of intensive, multi-agent chemotherapy regimens has dramatically improved disease outcomes, particularly in the pediatric setting (Pui et al., 2008). However, T-ALL patients whose disease is chemo-refractory or has relapsed remain a major unmet medical need (Litzow and Ferrando, 2015; Marks and Rowntree, 2017). Importantly, there are currently no targeted therapies available, and current immunotherapies have so far not proven effective in T-ALL (Ishitsuka et al., 2018; Ratner et al., 2018). Overall, this lack of effective treatment options necessitates the discovery of new therapeutic strategies and a better understanding of the disease biology (Litzow and Ferrando, 2015; Van Vlierberghe and Ferrando, 2012).

T-ALL is a biologically and genetically heterogeneous disease (for a recent review see Belver and Ferrando, 2016), reflecting developmental arrest and oncogenic transformation at different stages of thymocyte development (Ferrando et al., 2002). The NOTCH1 signaling pathway is critical for thymocyte development and is also a major oncogenic driver in T-ALL, with activating *NOTCH1* mutations detected in approximately 60% of T-ALL patients (Radtke et al., 2004; Weng et al., 2004). Despite its critical role in T-ALL, NOTCH1-targeting therapies have failed so far to produce strong anti-tumor responses, largely due to difficulties in finding acceptable therapeutic windows for these agents (Takebe et al., 2014; Wei et al., 2010).

The meaningful preclinical evaluation of novel therapeutic approaches requires the use of representative (of the clinical situation) and thus predictive mouse models; for the testing of immunotherapeutic drugs these models should provide a fully immunocompetent context. In fact, the lack of appropriately predictive preclinical models is frequently identified as a major reason for the high attrition rate in drug development (Hutchinson and Kirk, 2011). Existing preclinical T-ALL models are based on either the genetic manipulation of specific oncogenes or tumor suppressor genes, or the transplantation of human disease into immunodeficient host mice (García-Peydró et al., 2018; McCormack et al., 2013; McGuire et al., 1992; Pear et al., 1996). Recently, a spontaneously arising, thymus-transplantation-based mouse model of T-ALL was described (Martins et al., 2014), which gives rise to genetically distinct T-ALL cases recapitulating many of the key histologic and genetic features of the human disease. Here, we built on this model to develop it into a platform for preclinical testing. We improved the model by genetic modification of the murine *Notch1* locus to better align the spectrum of murine *Notch1* mutations to the human disease, thereby showing that recombination-activating gene (RAG)-mediated deletions in the 5′ region of the *Notch1* gene are the preferred mechanism to achieve *Notch1* activation in mice. Genetic deletion of Notch3 does not impair T-ALL development. Furthermore, we demonstrate the application of the model to the testing of targeted and immunotherapeutic agents in fully immunocompetent animals. In particular, we describe the effects of Notch1 inhibition and anti-PD-1 (programmed cell death 1) immune checkpoint blockade on T-ALL progression. Going forward, this enhanced, genetically heterogeneous model will be of great utility for the preclinical evaluation of novel therapeutic strategies against T-ALL.

## RESULTS

### Generation and characterization of the thymus-transplantation-based murine T-ALL model

**Fig. 1.**
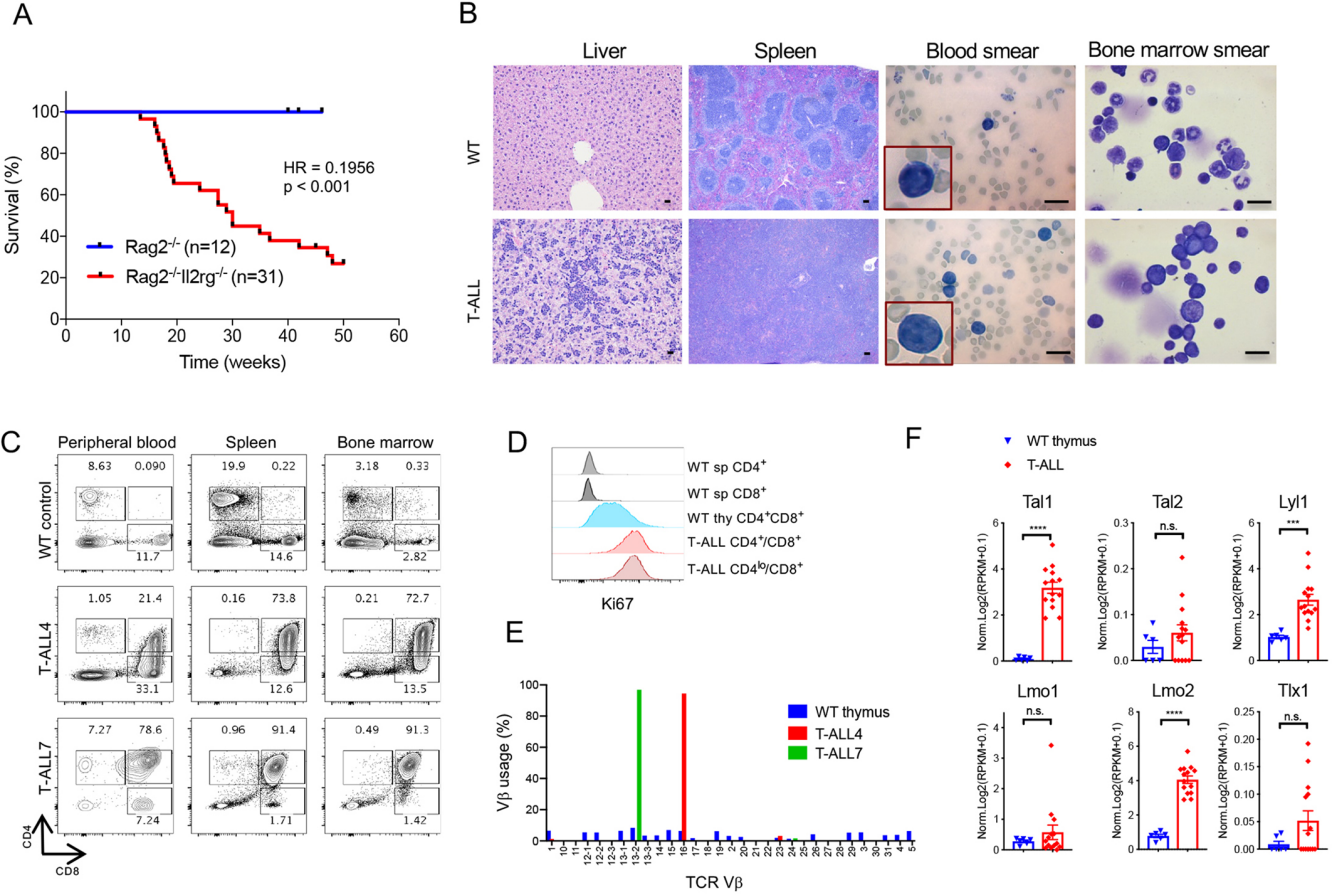
**Characterization of the murine thymus-transplantation-based T-ALL model.** (A) Survival curves of *Rag2*^−/−^*Il2rg*^−/−^ (DKO) and the control *Rag2*^−/−^ mice post-implantation of wild-type newborn thymus. Survival curves were compared by log-rank (Mantel-Cox) test. *P*-value and hazard ratio (HR) are indicated. (B) Representative H&E staining of liver and spleen, and Giemsa staining of bone marrow and peripheral blood smear from diseased T-ALL and wild-type (WT) mice. C57BL/6 mice were used as WT control. Scale bars: 10 mm. (C) Representative FACS plots of peripheral blood, spleen and bone marrow in two T-ALL mice (T-ALL4 and T-ALL7) and one WT control. (D) Ki67 staining of T-ALL cells (CD4^+^/CD8^+^ and CD4^lo^/CD8^+^ from spleen of a T-ALL mouse) and WT control T cells (CD4^+^ and CD8^+^ from spleen, and CD4^+^CD8^+^ from thymus of a C57BL/6 mouse). (E) TCR Vβ usage in normal WT thymus tissue compared to leukemic cells from T-ALL4 and T-ALL7. (F) Expression of oncogenic transcription factors in individual T-ALL cases compared to normal WT thymus. Each dot represents one mouse and bars represent mean±s.e.m. Groups were compared by one-way ANOVA with Dunnett's multiple-comparison test (***P<0.001; ****P<0.0001; n.s. not statistically significant).

To set up the thymus-transplantation-based mouse T-ALL model, newborn wild-type B6.Ly5.1 thymus (CD45.1^+^) was implanted under the kidney capsule of T-cell-progenitor-deficient CD45.2^+^ *Rag2^−/−^ Ιl2rg^−/−^* [double knockout (DKO)] mice, as described previously (Martins et al., 2014). DKO host mice exhibited signs of distress as early as 11 weeks post-transplant and succumbed to disease starting at 13 weeks post-thymus-implantation. More than 60% of transplanted DKO mice died within 1-year post-transplant, with about half of events occurring between 16 and 20 weeks (Fig. 1A). Thymocyte-progenitor proficient *Rag2^−/−^* host mice, used as controls in this study, did not exhibit any signs of leukemia development (Fig. 1A). Gross examination of transplanted DKO mice revealed significantly expanded thymic grafts as well as splenomegaly and pale bone marrow and liver, presumably due to the presence of leukemic cells (Fig. S1). Histological analysis demonstrated infiltration of neoplastic cells into liver and spleen, while blood and bone marrow smears confirmed the lymphoblastic morphology of the infiltrating cells (Fig. 1B). CD4^lo/+^CD8^+^ double-positive (DP) T cells were detected in peripheral blood, a hallmark of T-ALL, as well as in lymphoid tissues such as spleen and bone marrow (Fig. 1C). T-ALL cells, both CD4^+^CD8^+^ and CD4^lo^CD8^+^ populations, were highly proliferative (Fig. 1D). Individual T-ALL cases exhibited an immunophenotypic spectrum with varying expression of surface CD4, CD8, CD44 and CD25 (Fig. S2).

T-ALL obtained in individual mice was further characterized at the molecular level by whole-exome sequencing of unfractionated spleen cells (on average consisting of greater than 80% CD4^+/lo^CD8^+^ leukemic cells). Individual T-ALL cases exhibited variable, but generally low, numbers of point mutations and small insertions/deletions (Table 1), in line with the incidence of somatic mutation observed in human T-ALL (Ma et al., 2018). Copy number variation analysis identified large genomic lesions indicative of genomic instability in some T-ALL cases (Fig. S3A). Consistent with the high frequency of *NOTCH1* activating mutations in human T-ALL (Weng et al., 2004), gain-of-function (GOF) *Notch1* mutations were prominent in the spontaneous T-ALL model (Table 1). Notch1 PEST-domain mutations, increasing the stability of the Notch1 intracellular domain (N1-ICD), were found in 10 out of 19 (53%) T-ALL samples (Table 1). In addition, 10 out of 19 (53%) samples harbored 5′ deletion in the *Notch1* locus (Table 1, Fig. S3B), which was associated with the constitutive expression of intracellular Notch1 (Ashworth et al., 2010). Mutations in the Notch1 heterodimerization domain (HD) and the transactivation domain (TAD) were found at low frequency in T-ALL tumors: 11% and 5%, respectively (Table 1). Activating *Notch1* mutations in individual T-ALL cases were associated with the increased expression of the Notch1 target genes *c-Myc* and *Hes1* relative to normal T cells from C57BL/6 spleens (Fig. S4). The comparison of gene expression changes in the thymus-transplantation-based T-ALL model relative to a traditional N1-ICD-driven T-ALL model (Li et al., 2008) found overall similar trends in terms of gene changes relative to control. However, gene changes in the thymus-transplantation-based T-ALL model are much less uniform than in the transgenic N1-ICD model, supporting the notion that the thymus-transplantation-based T-ALL spontaneous model captures the molecular heterogeneity of the human T-ALL (Fig. S5).

**Table 1.**
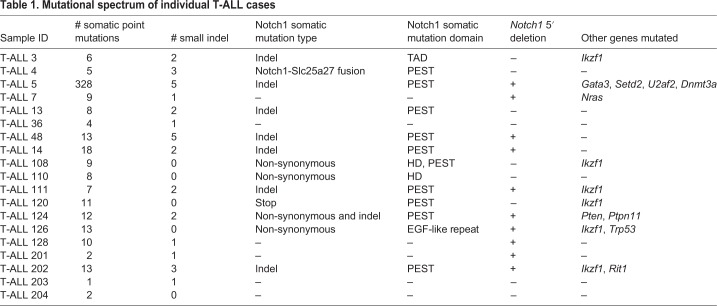
**Mutational spectrum of individual T-ALL cases**

Other genes frequently mutated in human hematological malignancies (Ley et al., 2010; Mullighan, 2013; Yoshida et al., 2011) were detected in murine T-ALL cases, including *Pten*, *Trp53*, *Ikzf1*, *Gata3*, *Setd2*, *U2af2*, *Ptpn11*, *Dnmt3a* and *Nras* (Table 1). RNA sequencing (RNAseq) and T-cell receptor (TCR) analysis confirmed monoclonality of leukemic disease (Fig. 1E) and detected elevated expression of canonical oncogenic transcription factors (Van Vlierberghe and Ferrando, 2012) in individual T-ALL cases, in particular *Tal1*, *Lyl1* and *Lmo2* (Fig. 1F). Collectively, these data demonstrate that the thymus-transplantation-based mouse T-ALL model recapitulates key features of the human disease, and that each case of mouse leukemia represents an individual disease, characterized by a unique set of phenotypic and genetic features.

### Humanization of the murine T-ALL *Notch1* mutational spectrum

**Fig. 2.**
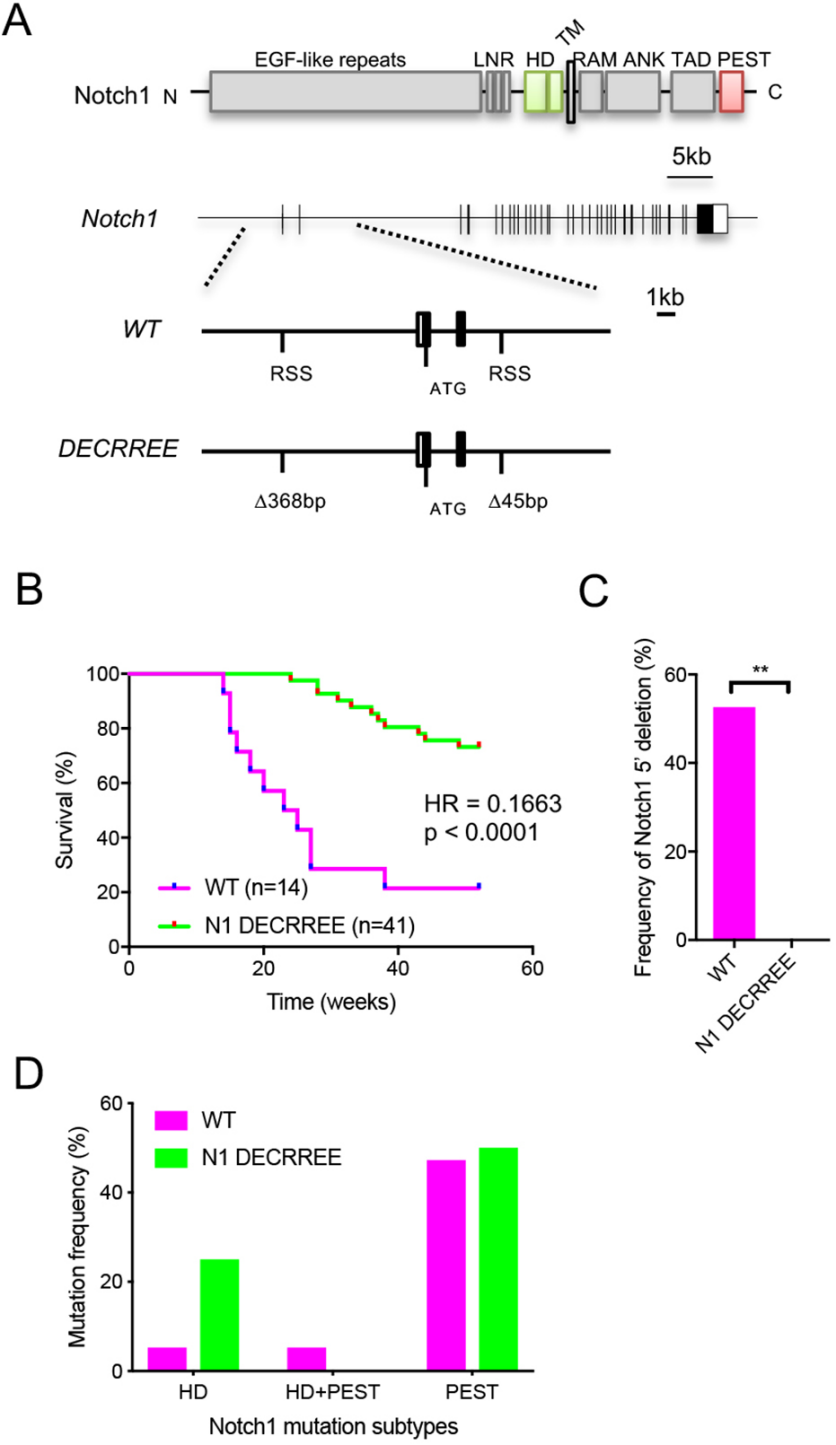
**Humanization of the murine T-ALL Notch1 mutational spectrum.** (A) Schematic of Notch1 protein structure and genomic locus. Insert depicts locations of recombination signal sequences (RSSs) in the 5′ end of the *Notch1* gene at positions −8131 and +3573, and the *Notch1*^DECRREE^ allele with selective deletion of RSSs. RSS deletion encompassed 368 bp and 45 bp, respectively. The start codon is indicated; coding exons are in black. (B) Survival curves of DKO mice transplanted with wild-type (WT) and *Notch1*^DECRREE^ thymus. Groups were compared by log-rank (Mantel-Cox) test. *P*-value and hazard ratio (HR) are indicated. (C) Frequency of *Notch1* 5′ deletions in WT- and *Notch1*^DECRREE^-derived T-ALL. Groups were compared by binomial test (**P<0.001). (D) Frequency of Notch1 HD- and PEST-domain mutations in WT- and Notch1^DECRREE^-derived T-ALL.

A distinctive feature of the spontaneous T-ALL model is the frequency of 5′ truncating mutations in the murine *Notch1* locus, resulting in translation initiation from a conserved methionine residue within the transmembrane domain and constitutive expression of N1-ICD (Ashworth et al., 2010); such mutations occur only very rarely in humans (Weng et al., 2004). These 5′ deletions appear to be RAG mediated and are associated with a series of cryptic RAG recombination signal sequences (RSSs) interspersed in the 5′ region of the *Notch1* gene (Ashworth et al., 2010) (Fig. 2A). We hypothesized that the genetic deletion of these ectopic RSSs would prevent aberrant 5′ *Notch1* recombination events, thus skewing the spectrum of murine *Notch1* mutations more towards that of the human disease. To that end, the RSS at position −8131, located in intron 1 upstream of the first coding exon of *Notch1*, and RSS at position +3573, within the third intron, were selectively deleted via genetic engineering in mouse embryonic stem (ES) cells (Fig. 2A, Fig. S6A). The resultant allele was termed *Notch1*^DECRREE^ (deletion of ectopic RAG recombinant sites).

Homozygous *Notch1*^DECRREE^ mice exhibited normal T-cell development (Fig. S6B) and responsiveness to *Notch1* signaling blockade, as evidenced by the reduced thymic mass and cellularity (Fig. S6C) consequent to the arrest in T-cell differentiation at the double-negative 1 (DN1) stage (Fig. S6D) (Billiard et al., 2011). To test the impact on leukemogenesis and mutational spectrum, neonatal thymi from homozygous *Notch1*^DECRREE^ mice were used as donor tissue in the T-ALL transplantation model. Compared to wild-type thymus-derived T-ALL, *Notch1*^DECRREE^ thymus-derived T-ALL development was markedly delayed with overall significantly decreased disease penetrance (Fig. 2B), indicating that RSS-RAG-mediated genomic 5′ deletions are a dominant mechanism of *Notch1* activation and resultant leukemogenesis in mice. Leukemic disease that did develop in *Notch1*^DECRREE^ mice was phenotypically similar to wild-type *Notch1*-derived T-ALL with regards to acuteness of disease, elevation of white blood cell counts, and cell-surface CD4/CD8 expression (data not shown). Molecular analysis confirmed that no *Notch1*^DECRREE^-derived T-ALL cases harbored 5′ *Notch1* genomic deletions (Fig. 2C), demonstrating that the RSS at positions −8131 and +3573 are indeed the main mediators of the ectopic RAG recombination events in the murine *Notch1* gene. Whole-exome and Sanger sequencing found activating *Notch1* mutations in 75% (6 out of 8) of *Notch1*^DECRREE^-derived T-ALL cases. Of those, heterodimerization (HD) domain mutations were present in 25%, similar to the *Notch1* HDD mutation frequency of 33% in human T-ALL patients (Grabher et al., 2006), compared to 11% in wild-type *Notch1* thymus-derived T-ALL (Fig. 2D). These findings suggest that the ablation of cryptic RAG-mediated 5′ recombination events in the murine *Notch1* locus does indeed change the range of resultant *Notch1* mutations towards the human spectrum and affect kinetics and penetrance of spontaneous T-ALL.

### Targeting of oncogenic *Notch1* signaling

NOTCH1 is a critical oncogenic driver in T-ALL, and activating *NOTCH1* mutations are present in around 60% of T-ALL patient tumors (Radtke et al., 2004; Weng et al., 2004). Gain-of-function *Notch1* mutations were detected at high frequency in our spontaneous T-ALL model (Table 1). *Notch1* mutations found in mouse T-ALL tumors fall into three main categories: (1) PEST-domain mutations, which increase the stability of N1-ICD, (2) 5′ deletions of the *Notch1* locus, which result in constitutive expression of N1-ICD, and (3) Notch1 HD domain mutations, which increase Notch1 susceptibility to ligand-independent, proteolytic activation (Malecki et al., 2006), and which were detected with increased frequencies in murine T-ALL cases derived from *Notch1*^DECRREE^ thymus. Based on their respective mechanisms of Notch1 activation, these three classes of Notch1 mutations are thought to exhibit different sensitivities to the two main types of clinical Notch1 inhibitory monoclonal antibodies (mAbs): ligand-blocking (Noguera-Troise et al., 2006) and negative regulatory region (NRR)-targeting (Gordon et al., 2009) mAb. Notch1 PEST-domain mutations are dependent on ligand binding to elicit signaling activity and would be sensitive to both types of inhibitory mAb. *Notch1* 5′ deletions would be resistant to both types of inhibitory antibodies, whereas Notch1 HD-domain mutation-mediated signaling would be specifically blocked by mAb targeting the NRR.

Taking advantage of the thymus-transplantation-based T-ALL system, we set out to test this notion experimentally. First, using an *in vitro* OP9/OP9-DLL1 co-culture system, we confirmed that T-ALL cases characterized by Notch1 PEST-domain mutations (T-ALL4, T-ALL13) required an exogenous source of Notch1 ligands for growth, whereas T-ALL cells with 5′ *Notch1* deletions (T-ALL48, T-ALL5) grew independent of ligand expression (Fig. S7). T-ALL cases with *Notch1* 5′ deletions and PEST domain mutations were sensitive to gamma secretase inhibitor (GSI) treatment *in vitro*. Interestingly, T-ALL7, which carries a *Notch1* 5′ deletion and an *Nras* mutation, was resistant to GSI treatment, suggesting that leukemic cell proliferation is driven by Nras in this model (Fig. S8).

**Fig. 3.**
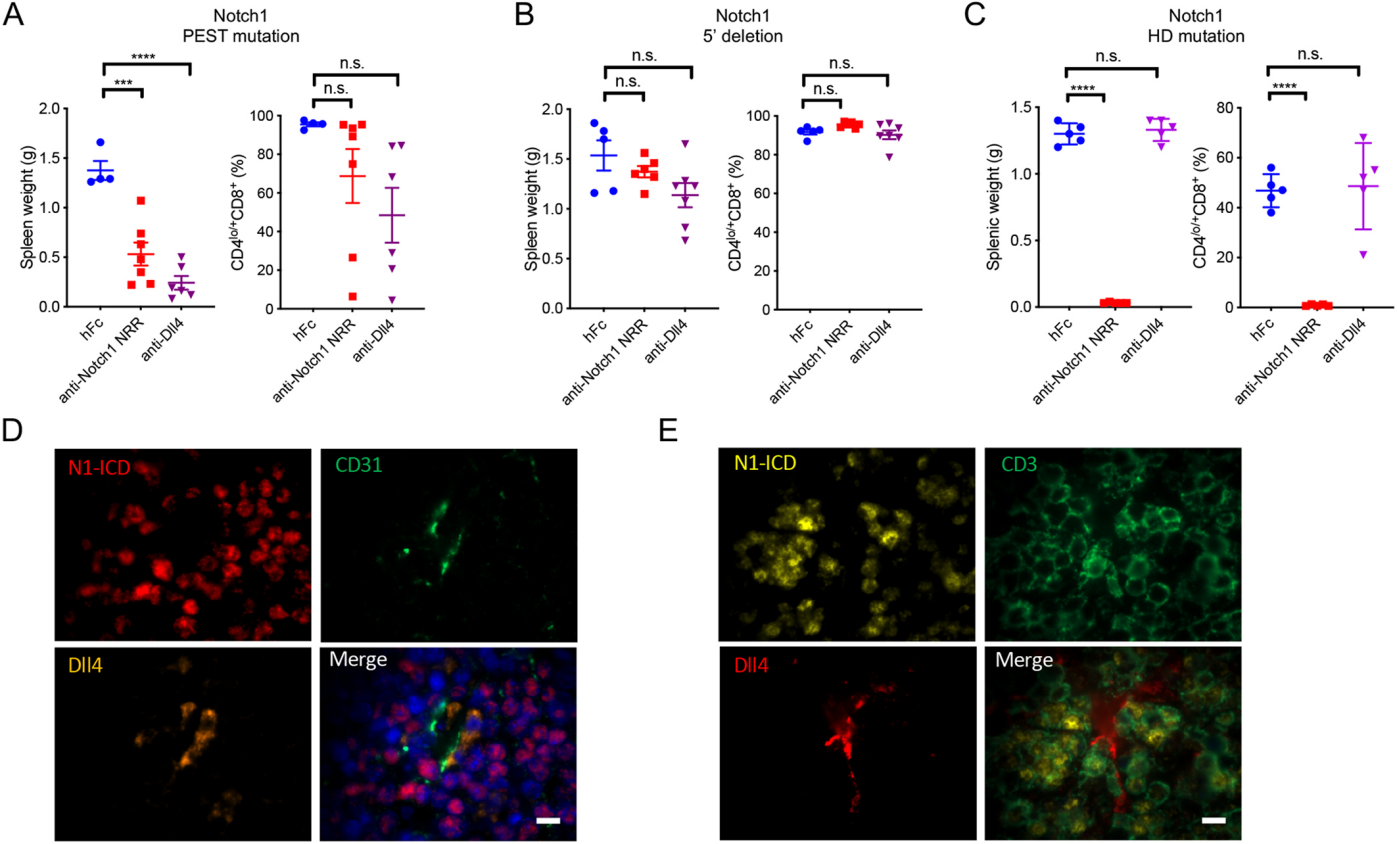
**Notch1 mutational status determines T-ALL treatment response to therapeutic monoclonal antibodies.** (A-C) Spleen weight and T-ALL burden (percentage of CD4^lo/+^CD8^+^ leukemic cells) in the spleen in secondary DKO hosts transplanted with T-ALL4 (with Notch1 PEST mutation; A), T-ALL48 (with *Notch1* 5′ deletion; B) and T-ALL DECRREE2 (with Notch1 HD mutation; C). Mice were treated with hFc control (10 mg/kg body weight, twice a week), Notch1 NRR-targeting antibody (10 mg/kg body weight, twice a week) or Dll4-blocking antibody (5 mg/kg body weight, once a week). Each dot represents one mouse and bars represent mean±s.e.m. Treatment groups were compared by one-way ANOVA with Dunnett's multiple-comparison test (***P<0.001; ****P<0.0001; n.s. not statistically significant). (D) Immunofluorescent staining for Notch1-ICD (red), endothelial marker CD31 (green) and Dll4 (orange) in spleen of T-ALL4-bearing mice. Nuclear DAPI staining (blue) was merged with the other markers in the bottom right panel. (E) Immunofluorescent staining of Notch1-ICD (yellow), T-cell marker CD3 (green) and Dll4 (red) in spleen of T-ALL4-bearing mice. Nuclear DAPI staining (blue) was merged with the other markers in the bottom right panel. Scale bars: 10 mm.

We next sought to test the sensitivity of individual T-ALL cases representing the three categories of Notch1 mutations to inhibitory mAb treatment *in vivo*. For treatment studies, we serially *in vivo* passaged leukemic cells harvested from spleen of primary hosts into secondary immunodeficient DKO hosts. Disease take rate was 100% in secondary DKO recipients, with accelerated tumor growth (median survival time between 25 and 56 days) (Fig. S9). In particular, primary disease from T-ALL4 (Notch1 PEST-domain mutation), T-ALL48 (*Notch1* 5′ deletion) and T-ALL DECRREE2 (*Notch1* HD domain mutation) was transplanted into secondary DKO host mice. When peripheral CD4^lo/+^CD8^+^ leukemic T-cell burden reached 0.5%, mice were treated with a Dll4-blocking mAb (5 mg/kg body weight, once a week), a Notch1-NRR-targeting mAb or control antibody (10 mg/kg body weight, twice weekly). Terminal analysis was performed once mice displayed signs of overt distress. For T-ALL4 (Notch1 PEST-domain mutation), splenomegaly was evident in control-treated animals. However, spleens from mice treated with the Dll4-blocking or the anti-Notch1-NRR antibody had essentially normal spleen weights (Fig. 3A), and peripheral leukemic disease burden was markedly reduced in several anti-Dll4- and anti-Notch1-NRR-treated animals (Fig. 3A). Immunofluorescence analysis of spleens from T-ALL4 demonstrated active Notch1 signaling (N1-ICD^+^) in close proximity to Dll4-positive endothelial cells (Fig. 3D,E), suggesting paracrine signaling interactions and supporting a potential dominant role for Dll4 in promoting oncogenic Notch1 signaling. In contrast to the treatment effects in T-ALL4, Notch1-NRR-targeting or Dll4-blocking mAb did not significantly impact splenic or peripheral disease burden in mice bearing T-ALL48 (*Notch1* 5′ deletion) (Fig. 3B). Lastly, tumor growth in mice transplanted with T-ALL DECRREE2 (HD-domain mutation) was completely inhibited by the treatment with the NRR-targeting anti-Notch1 mAb, but unaffected by Dll4 ligand blockade (Fig. 3C). Collectively, these results demonstrate the applicability of the thymus-transplantation-based T-ALL platform to the preclinical testing of targeted therapies, and further strengthen the experimental framework for the rational mAb-based targeting of Notch1 signaling based on Notch1 mutational status.

### Genetic ablation of Notch3 does not impact T-ALL development

**Fig. 4.**
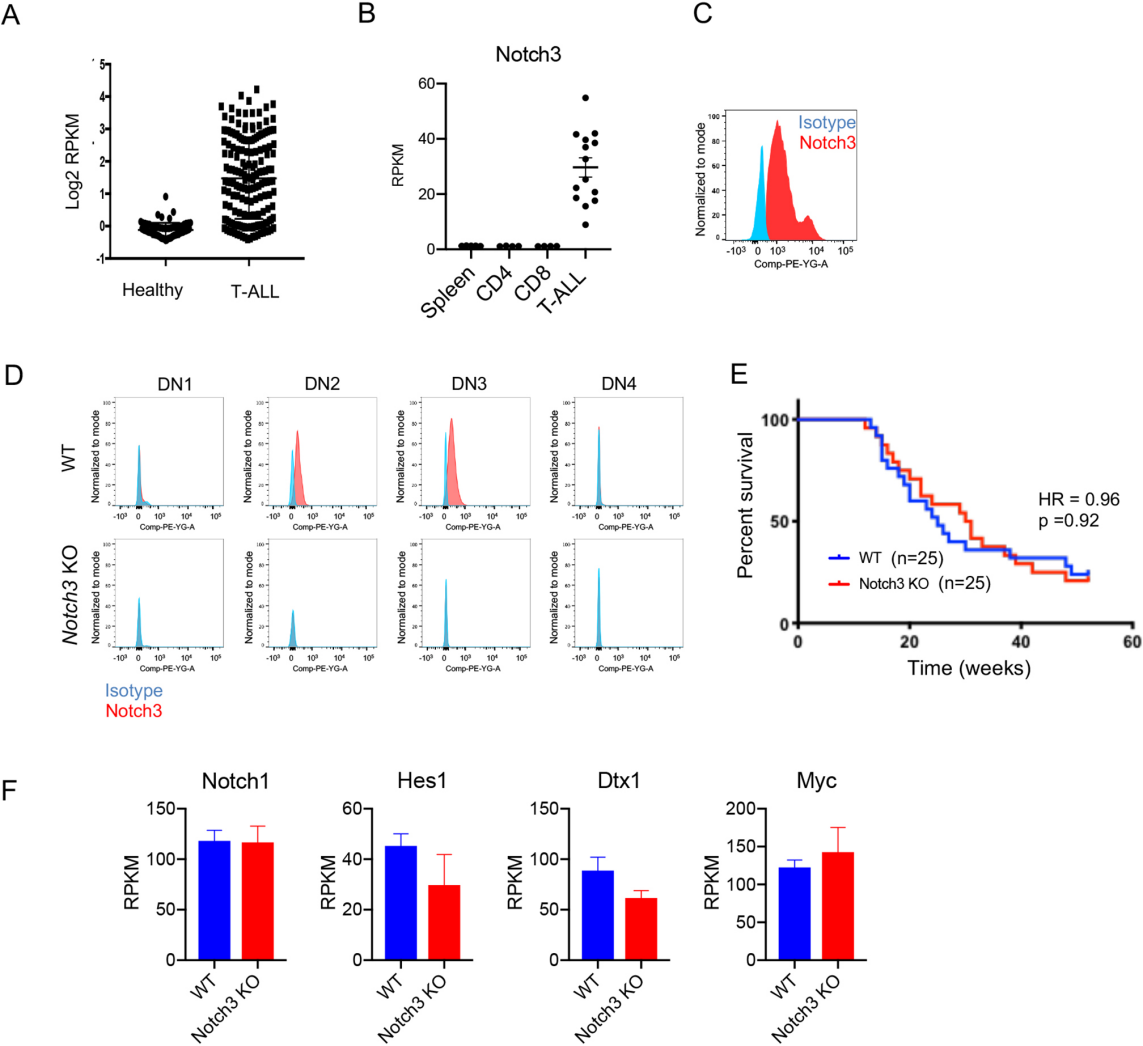
**Genetic ablation of *Notch3* does not impair T-ALL development in** **the** **thymic transplant model.** (A) *NOTCH3* RNA expression in healthy human bone marrow and T-ALL patient bone marrow samples from the Oncomine Haferlach leukemia dataset, which includes 174 T-ALL cases and 74 non-leukemia and healthy bone marrow control cases. RPKM, reads per kb per million mapped reads. (B) Expression of *Notch3* RNA in individual T-ALL cases compared to normal spleen from DKO mice and splenic C57BL/6 CD4^+^ and CD8^+^ cells. Each dot represents one mouse and bars represent mean±s.e.m. (C) Cell surface expression of Notch3 in a representative T-ALL case. T-ALL spleen cells were stained for either Notch3 or isotype control. (D) Notch 3 cell surface expression at the double negative (DN) stages of thymocyte development in wild-type and *Notch3*-KO thymi. (E) Survival curves of DKO mice transplanted with wild-type or *Notch3*-KO thymus. Survival curves were compared by log-rank (Mantel-Cox) test. *P*-value and hazard ratio (HR) are indicated. (F) Expression of *Notch1* and Notch1 target genes in wild-type- and *Notch3*-KO-derived T-ALL spleen cells. Mean±s.e.m. are plotted.

Although GOF mutations in *NOTCH3* are infrequent in T-ALL, active NOTCH3 signaling, correlating with overall high *NOTCH3* expression, was recently detected at higher than expected frequencies in T-ALL patient samples and patient-derived xenograft (PDX) models (Bernasconi-Elias et al., 2016). This suggests functional involvement of Notch3 in T-ALL development uncoupled from NOTCH3 mutational status. The oncogenic potential of Notch3 has been demonstrated in T-ALL mouse models (Bellavia et al., 2000). We confirmed *NOTCH3* upregulation in human T-ALL patient samples (Fig. 4A) and found that Notch3 expression was upregulated at both the RNA and surface protein level in the spontaneous mouse T-ALL models (Fig. 4B,C). Sequencing analysis did not detect activating mutations in *Notch3* (data not shown). To elucidate whether *Notch3* upregulation contributes to leukemogenesis in the spontaneous T-ALL model, we generated *Notch3* knockout (KO) mice. Loss of Notch3 protein expression in the KO mice was confirmed by flow cytometry analysis of thymocyte-progenitor populations at DN2 and DN3 stages (Fig. 4D). Consistent with published reports (Krebs et al., 2003; Talora et al., 2006), Notch3 ablation did not impair normal T-cell development (Fig. S10). Next, thymic lobes from the *Notch3* KO animals were transplanted under the kidney capsule of immunodeficient mice to promote T-ALL development. Disease onset and frequency in *Notch3* KO thymus-derived T-ALL was indistinguishable from wild-type controls (Fig. 4E), indicating that Notch3 activity is not critical for T-ALL development in this model. The potential role for oncogenic Notch3 signaling may be masked by the propensity of this model to develop *Notch1* GOF mutations. Consistent with this possibility, we observed multiple ligand-independent *Notch1* mutations in *Notch3^−/−^* tumors that are sufficient to drive leukemogenesis of T-ALL (data not shown), as well as activation of the Notch1 signaling pathway. Notch1 itself and Notch1 target genes (*Hes1*, *Dtx1*, *cMyc*) were upregulated at similar levels between wild type and *Notch3*-KO-derived T-ALL (Fig. 4F). Overall, our results show that Notch3 ablation does not affect T-ALL development.

### Therapeutic testing of PD-1 immune checkpoint blockade

To enable the preclinical evaluation of immunomodulatory therapies, we tested whether primary leukemic disease that developed in immunodeficient DKO mice could be serially *in vivo* transplanted into syngeneic, immunocompetent C57BL/6 hosts. Disease burden in syngeneic C57BL/6 (CD45.2^+^) mice was monitored by the congenic marker CD45.1. Individual primary T-ALL cases showed more variable tumor take rates when engrafted into immunocompetent mice and slower growth kinetics compared to immunodeficient recipients (Fig. S11), suggesting immunoediting (Mittal et al., 2014). Analysis of somatic mutation and variant allele frequency in primary versus secondary T-ALL in immunocompetent mice detected unique changes in mutation burden for different secondary T-ALL, suggesting individualized tumor evolution (Fig. S12).

**Fig. 5.**
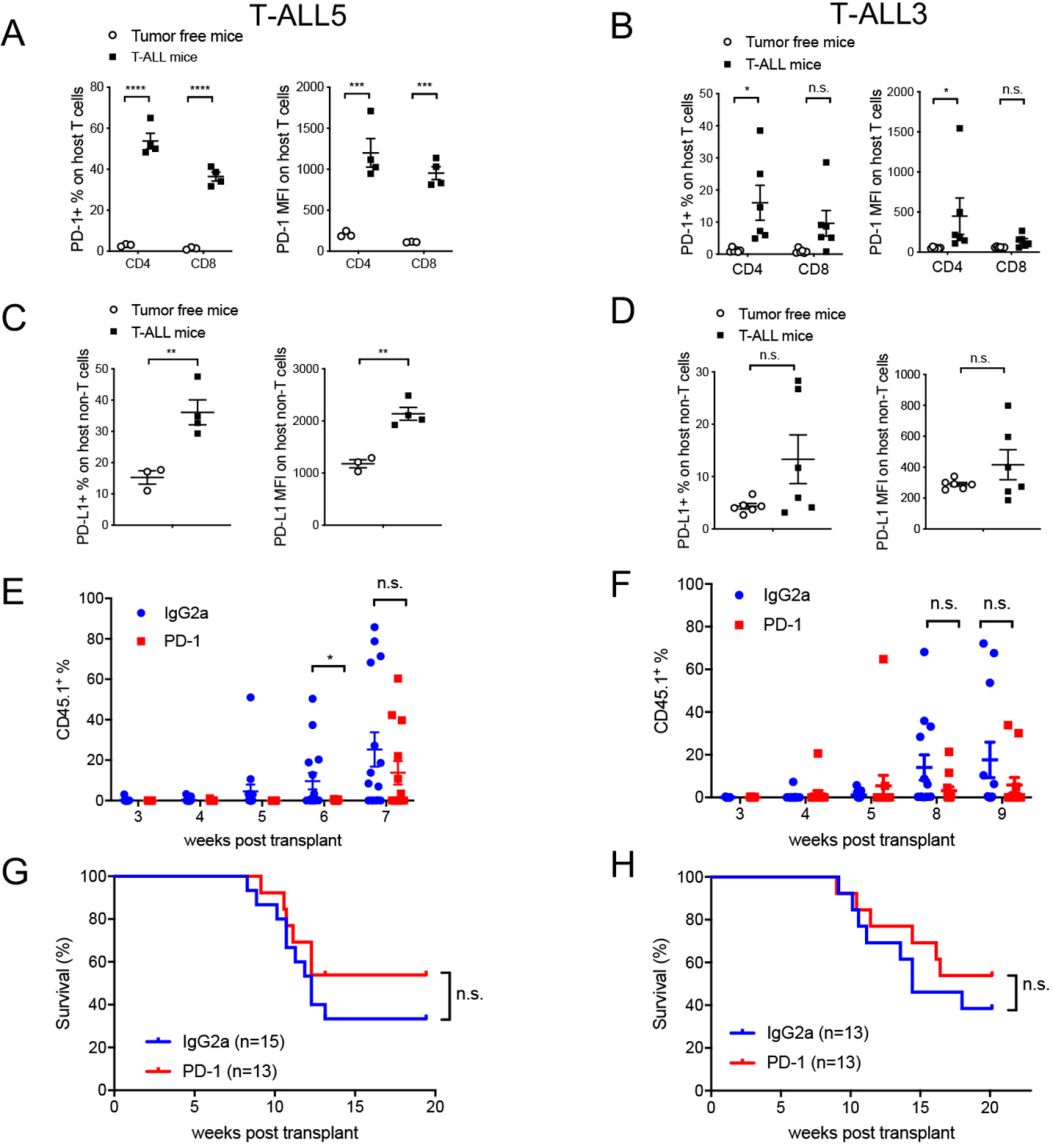
**PD-1 blockade alone lacks anti-tumor activity against T-ALL tumors.** (A-D) Analysis of C57BL/6 mice with established T-ALL5 and T-ALL3. (A,B) PD-1 expression [frequency and mean fluorescent intensity (MFI) levels] in peripheral blood host T cells (CD4^+^ and CD8^+^) was analyzed by FACS in T-ALL5 (A)- and T-ALL3 (B)-bearing mice relative to tumor-free control mice. (C,D) PD-L1 expression (frequency and MFI levels) in peripheral blood host non- T-cells (CD4^−^ and CD8^−^) by FACS in T-ALL5 (C)- and T-ALL3 (D)-bearing mice relative to tumor-free control mice. (E,F) T-ALL5 (E)- and T-ALL3 (F)-bearing mice were treated with either IgG2a isotype control or anti-PD-1 antibody. Donor-derived T-ALL cells (CD45.1^+^) in peripheral blood were monitored over time. (G,H) Survival curves of treated mice bearing T-ALL5 (G) and T-ALL3 (H), corresponding to E and F, respectively. Each dot represents one mouse and bars represent mean±s.e.m. Treatment groups were compared by one-way ANOVA with Dunnett's multiple-comparison test (A-F). Survival curves were compared by log-rank (Mantel-Cox) test. *P<0.05; **P<0.01; ***P<0.001; ****P<0.0001; n.s. not statistically significant.

Analysis of host T cells demonstrated significant PD-1 upregulation on normal host T cells, both CD4 and CD8, in syngeneic mice bearing T-ALL5 relative to non-tumor-bearing C57BL/6 controls (Fig. 5A) and a trend toward increased PD-1 expression in mice bearing T-ALL3 (Fig. 5B). While programmed cell death 1 ligand 1 (PD-L1) expression was not detected on leukemic T cells (data not shown), PD-L1 was found to be expressed in a significant fraction of non-T-cell host immune cells, presumably myeloid cells, in T-ALL5-bearing C57BL/6 mice relative to non-tumor-bearing wild-type mice (Fig. 5C); a more moderate increase in PD-L1 expression was seen for T-ALL3 (Fig. 5D). The above findings prompted us to test the efficacy of anti-PD-1 immune checkpoint blockade in this model. We initially selected T-ALL5 for this analysis, which was characterized by an unusually high number of somatic mutations compared to the other mouse T-ALL cases and also to the human disease (Table 1). Treatment with PD-1-blocking mAb (rat anti-mouse clone RMP1-14) or isotype control was initiated 3 weeks post-transplantation when peripheral CD45.1^+^ leukemic burden was around 0.2%. Twice-weekly mAb treatment at 10 mg/kg body weight was maintained for the duration of the study. Treatment with the PD-1-blocking mAb resulted in transient control of tumor growth; however, breakthrough leukemic disease was observed by week 7 post-transplant (after 4 weeks of treatment) (Fig. 5E). Correspondingly, anti-PD-1-treatment did not result in statistically significant survival benefits (Fig. 5G). Next, we tested the activity of PD-1 blockade in T-ALL3, a model with low tumor mutational burden (Table 1), similar to what is typically observed in T-ALL patients. Again, treatment was initiated 3 weeks post-transplantation, when peripheral disease burden was around 0.2%. Anti-PD-1 mAb treatment did not control tumor growth or improve survival in this model (Fig. 5F,H). Analysis of host T-cell frequencies in T-ALL-bearing host mice at the start of treatment were comparable to those observed in non-tumor-bearing C57BL/6 mice for both T-ALL models (#5 and #3) (Fig. S13); thus, the lack of adequate host T-cell levels cannot account for the absence of treatment effects. Overall, these findings demonstrate that the thymus-transplantation-based T-ALL model can be applied to the preclinical testing of immunotherapies in fully immunocompetent mice. Our data show that PD-1 blockade alone lacks significant anti-tumor activity against murine T-ALL.

## DISCUSSION

T-ALL is an aggressive hematological cancer derived from the malignant transformation of T-cell progenitors. Outcomes remain poor for T-ALL patients with primary resistance to standard-of-care chemotherapy or with disease relapse. Unfortunately, major advances in our understanding of T-ALL disease biology have thus far not been translated into effective targeted therapies. The lack of effective next-generation therapies in T-ALL indicates that more relevant (i.e. more representative and predictive) models are needed to identify and validate targets and treatment approaches for this challenging disease. Martins et al. recently described a thymus-transplantation-based T-ALL model that uniquely recapitulated the key histopathological and genetic features of the human disease (Martins et al., 2014). This model is based on the initial observation that transplantation of wild-type neonatal thymus into severely immunodeficient mice results in long-term donor-autonomous T-cell development (Martins et al., 2012) that was associated with the spontaneous, i.e. wild-type donor-tissue-derived, development of T-ALL (Martins et al., 2014). Importantly, each case of mouse T-ALL harbored a unique set of genetic lesions, representing individual disease, contrasting with genetically engineered mouse models of T-ALL, which typically involve manipulating specific tumor suppressors or oncogenes (McCormack et al., 2013; McGuire et al., 1992; Pear et al., 1996). Uncoupling T-ALL development from investigator-directed mutations offers a potentially less-biased approach to identify key factors driving disease pathogenesis and to assess treatment responses. Our analysis confirmed the striking recapitulation of human disease features in the thymus-transplantation-based murine T-ALL model, including immunophenotype, genetic heterogeneity, frequency of somatic mutations, preponderance of *Notch1* mutations, presence of mutations in known hematologic cancer genes and elevation of oncogenic transcription factor expression.

The high relevance of this T-ALL model to the human disease prompted us to adapt it to the preclinical testing of targeted and immunotherapies. To this end, we pursued four lines of investigation: (1) genetic engineering of the T-ALL model to humanize the spectrum of the murine *Notch1* mutations; (2) the preclinical testing of Notch1-targeting therapeutic antibodies correlative to the Notch1 mutational status; (3) the assessment of Notch3 signaling contribution to leukemogenesis; and (4) the establishment of leukemic disease in fully immunocompetent, syngeneic host mice for the therapeutic testing of immune checkpoint blockade.

A distinctive feature of the spontaneous T-ALL model is the frequency of 5′ truncating mutations in the murine *Notch1* locus; this mutation occurs only very rarely in humans (Weng et al., 2004). The generation of these 5′ deletions is associated with a series of cryptic RAG RSSs in the 5′ region of the *Notch1* gene (Ashworth et al., 2010). Through deletion of RSS via genetic engineering (*Notch1*^DECRREE^ allele) in the context of the thymus-transplantation model, we were able to demonstrate that the RSSs at positions −8131 and +3573 are the principal mediators of these cryptic RAG recombination events, thus providing experimental evidence for a concept first described by Ashworth et al. (2010). Owing to the absence of 5′ *Notch1* deletions, leukemogenesis was severely impaired following transplantation of *Notch1*^DECRREE^ thymus, highlighting this mechanism as a preferred pathway to achieve Notch1 pathway activation in murine T-ALL. Concomitant to absence of 5′ *Notch1* deletions, the analysis of *Notch1*^DECRREE^-derived T-ALL suggested an increase in Notch1 HD-domain mutations and thus the alignment of the mouse and human *Notch1* mutational spectrums, although a greater sample size is needed to unequivocally support this notion.

Taking advantage of the spectrum of *Notch1* mutations observed in the thymus-transplantation model, we tested the anti-tumor activity of therapeutic mAb against leukemias characterized by *Notch1* 5′ deletions, HD- and PEST-domain mutations. The two classes of clinical Notch1 inhibitory mAbs block Dll4 ligand binding (Noguera-Troise et al., 2006) and target the negative regulatory region (NRR) (Wu et al., 2010), respectively. In line with previous findings (Aste-Amézaga et al., 2010), we found differential sensitivity of leukemias to these blockers based on Notch1 mutational status, with *Notch1* 5′-deletion-harboring tumors being resistant to either mAb, Notch1 PEST-domain mutant tumors being sensitive to both blockers, and tumors characterized by a Notch1 HD-domain mutation exhibiting selective responsiveness to the NRR-targeting antibody. Notch1 PEST-domain mutations have been identified as weaker oncogenic drivers in mouse T-ALL models and have been shown to cooperate with other genetic lesions to promote leukemogenesis (Chiang et al., 2008). Given the genetic heterogeneity of the thymus-transplantation T-ALL model, it would be interesting to identify genetic events that may cooperate with Notch1 PEST-domain mutations. Nevertheless, the robust response of the mutant-PEST-domain T-ALL model to Notch1-targeting mAb clearly supports the notion of Notch ‘addiction’ (Chiang et al., 2008).

Beyond further strengthening the framework for the rational targeting of *Notch1* mutant tumors based on mutational profile, this study demonstrates the general feasibility of incorporating germline genetic engineering into the model. To further illustrate this point, we have generated *Notch3* KO mice to assess the contribution of Notch3 to T-ALL development. Published results have demonstrated an oncogenic role for Notch3 in preclinical T-ALL models and treatment efficacy for Notch-signaling inhibitors in T-ALL cell lines wild type for *Notch1* but harboring *Notch3* GOF mutations (Bellavia et al., 2000; Choi et al., 2017). *Notch3*-deficient thymus tissue gave rise to T-ALL with similar efficiency as wild-type controls, indicating that elevated Notch3 levels do not contribute to disease. We hypothesize that this lack of effect is due to the high propensity of this model to develop *Notch1* GOF mutations. Indeed, we found activating *Notch1* mutations and active Notch1 signaling in *Notch3*-KO-derived T-ALL. This genetic functionalization opens up numerous possibilities to derive additional value. Along the lines of the study presented here, additional types of humanizations can be introduced. This may include the humanization of regulatory regions, or specific genes or gene regions, such as exons encoding the extracellular portion of a membrane protein. One obvious benefit of the latter approach would be that clinical candidate antibodies that often do not bind to the mouse ortholog can be directly tested in the murine T-ALL model.

To evaluate immune-targeted therapeutic approaches, we established the thymus-based T-ALL model in immunocompetent, syngeneic hosts via serial passaging into C57BL/6 mice. Although we detected substantial expression of PD-L1 in a significant fraction of non-T-cell host immune cells and of PD-1 in host T cells, which seemed to correlate with tumor mutational burden (TMB), we did not observe pronounced anti-tumor effects for the treatment with PD-1-blocking antibodies. Transient tumor control was observed in a T-ALL case with unusually high mutational load (T-ALL5); however, the treatment effect was lost after continued dosing beyond week 4 of treatment. This transient effect could be explained by the generation of therapeutic antibody-neutralizing antibodies, since the anti-PD-1 mAb used in the study was derived from rat; the overall kinetics of loss of anti-tumor activity is consistent with this mechanism. In a T-ALL case with low TMB (T-ALL3), similar to the levels found in human T-ALL, anti-PD-1 treatment did not result in tumor control. This finding is consistent with the clinical experience with immune checkpoint blockers in T-cell malignancies (Ishitsuka et al., 2018; Ratner et al., 2018) and the general concept that TMB is a major determinant of anti-PD-1 treatment efficacy (Hellmann et al., 2018). Having established a fully immunocompetent platform for therapeutic testing, future steps will focus on the assessment of rational combination therapies, among others the combination of PD-1 blockade with modalities promoting tumor immunogenicity, such as cancer vaccines (Grenier et al., 2018) or immunogenic cell-death-promoting chemotherapy (Galluzzi et al., 2017).

As with any model, the thymus-transplantation-based T-ALL model is not a perfect representation of the human disease; certain aspects of human T-ALL are not recapitulated. For instance, the early-T-cell progenitor (ETP) subtype of T-ALL, typically associated with activating *IL7R* mutations (Zhang et al., 2012), does not appear to be represented in the model. Most likely, this is a function of transformation occurring at later stages of thymocyte development in this model. In this context, it would be interesting to investigate whether the genetic ablation of certain key factors of thymocyte development could shift the phenotype of resultant T-ALL to a more immature phenotype. Nevertheless, this model uniquely represents key features and the genetic heterogeneity of the human disease. Coupled with the ability for genetic functionalization and therapeutic testing in fully immunocompetent backgrounds, going forward this platform will be of great use for the preclinical identification and validation of new drug targets and treatment approaches in T-ALL.

## MATERIALS AND METHODS

### Mice and transplantation procedures

All mouse experiments were approved by Regeneron Pharmaceuticals. Recipient mice were 6- to 8-week-old *Rag2^−/−^* and *Rag2^−/−^Il2rg^−/−^* (DKO) BALB/c mice purchased from Taconic. Thymus donors were B6.SJL-Ptprc^a^ Pep3^b^/BoyJ (H-2^b^; CD45.1) (termed B6.Ly5.1). One thymus lobe from 1-day-old mice was grafted under the kidney capsule of one recipient. For secondary T-ALL transplant, 1×10^5^ to 1×10^6^ splenocytes were transplanted by retro-orbital intravenous injections into DKO or C57BL/6 mice as indicated. *Notch1*^DECRREE^ mice were generated by deleting two RAG recombination sites, located at position −8131 in intron 1 upstream of the first coding exon, and at position +3573 within the third intron, respectively. *Notch3^−/−^* mice were generated by inserting a β-galactosidase gene in frame into the first coding exon of *Notch3* gene using VelociGene^®^ technology.

### Flow cytometry

Tissues were harvested and single-cell suspensions were prepared in PBS with 3% fetal bovine serum. Antibody staining and FACS analysis was performed as previously described (Gao and Aifantis, 2009). The following monoclonal antibodies from BioLegend were used: anti-mCD45.1/Ly5.1 (A20), anti-mCD45.2/Ly5.2 (104), anti-mCD4 (RM4-5), anti-mCD8a (53-6.7), anti-mCD44 (IM7), anti-mCD25 (PC61), anti-mNotch3 (HMN3-133), anti-mPD-1 (29F.1A12), anti-mPD-L1 (10F.9G2) and anti-mCD45 (30F11). Anti-Ki67 (B56) was purchased from eBioscience. Antibodies were directly coupled to FITC, PE, PerCpCy5.5, PECy7, APC, APCCy7, Alexa Fluor 700 and Pacific Blue. Data were acquired on BD Fortessa and analyzed by FlowJo.

### Histological analyses

Mice were sacrificed and necropsied; dissected tissue samples were fixed for 24 h in 4% paraformaldehyde, dehydrated, and embedded in paraffin. Paraffin blocks were sectioned at 4 µm and stained with H&E.

### Exome and RNA sequencing

Whole-exome capture was performed by using the Agilent Sure-Select Mouse All Exon 50 Mb kit, followed by 100 bp paired-end sequencing on the Illumina HiSeq 2000 platform. For RNAseq, total RNA was converted to mRNA libraries using KAPA Stranded mRNA-Seq kits from KAPA Biosystems following the manufacturer's protocols. Libraries were sequenced by 100 bp paired-end reads on the Illumina HiSeq 2000 with ∼75-million reads per sample. See supplemental methods for details of the analysis pipeline.

### Antibodies

Research-grade, function-blocking antibodies to mouse Notch1 and Dll4 (Kuhnert et al., 2015) were generated using VelociImmune^®^ technology. Anti-Notch1 (10 mg/kg body weight, twice a week), Dll4 (5 mg/kg body weight, once a week) and control antibodies (10 mg/kg body weight, twice a week) were administered intraperitoneally (IP). Anti-PD-1 (RMP1-14) and isotype control rat IgG2a (2A3) were purchased from BioXcell and administered IP at 10 mg/kg body weight twice a week starting at 3 weeks post-transplantation. For immunofluorescent staining, anti-CD31 (ab28364, Abcam), anti-N1-ICD (D3B8, Cell Signaling Technology) and anti-Dll4 (AF1389, R&D Systems) were used.

### Statistical analysis

Data are presented as mean±s.e.m. Groups were compared using one-way ANOVA with Dunnett's multiple comparison test or Binomial test, as indicated in figure legends. Survival curves were compared by Log-rank (Mantel-Cox) test. *P* values are indicated in figure legends.

## Supplementary Material

Supplementary information
