# Supplementary material for: Therapeutic targeting of Notch signaling and immune checkpoint blockade in a spontaneous, genetically heterogeneous mouse model of T-cell acute lymphoblastic leukemia
Source: Dis Model Mech. 2019 Sep 16;12(9):dmm040931. doi: 10.1242/dmm.040931 (PMC6765191; doi:10.1242/dmm.040931)
Supplement: Supplementary information [file dmm-12-040931-s1.pdf]

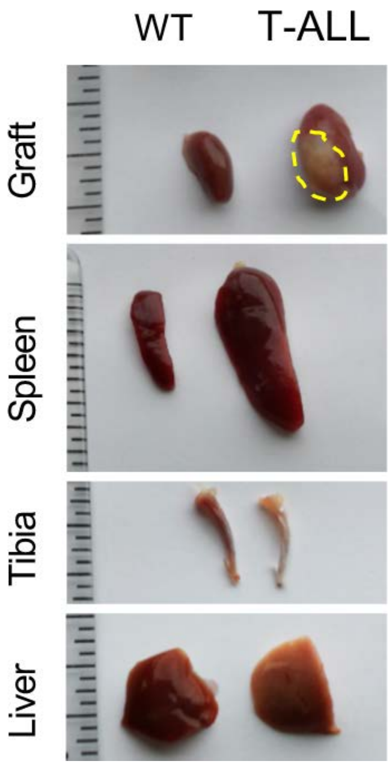

Fig. S1. Macroscopic features of murine T-ALL derived via thymic transplantation.
Representative images of an expanded thymic graft under kidney capsule (outlined in yellow), spleen, tibia, and liver from diseased T-ALL and wild type (WT) mice. C57Bl/6 mice were used as WT control.

Disease Models & Mechanisms • Supplementary information

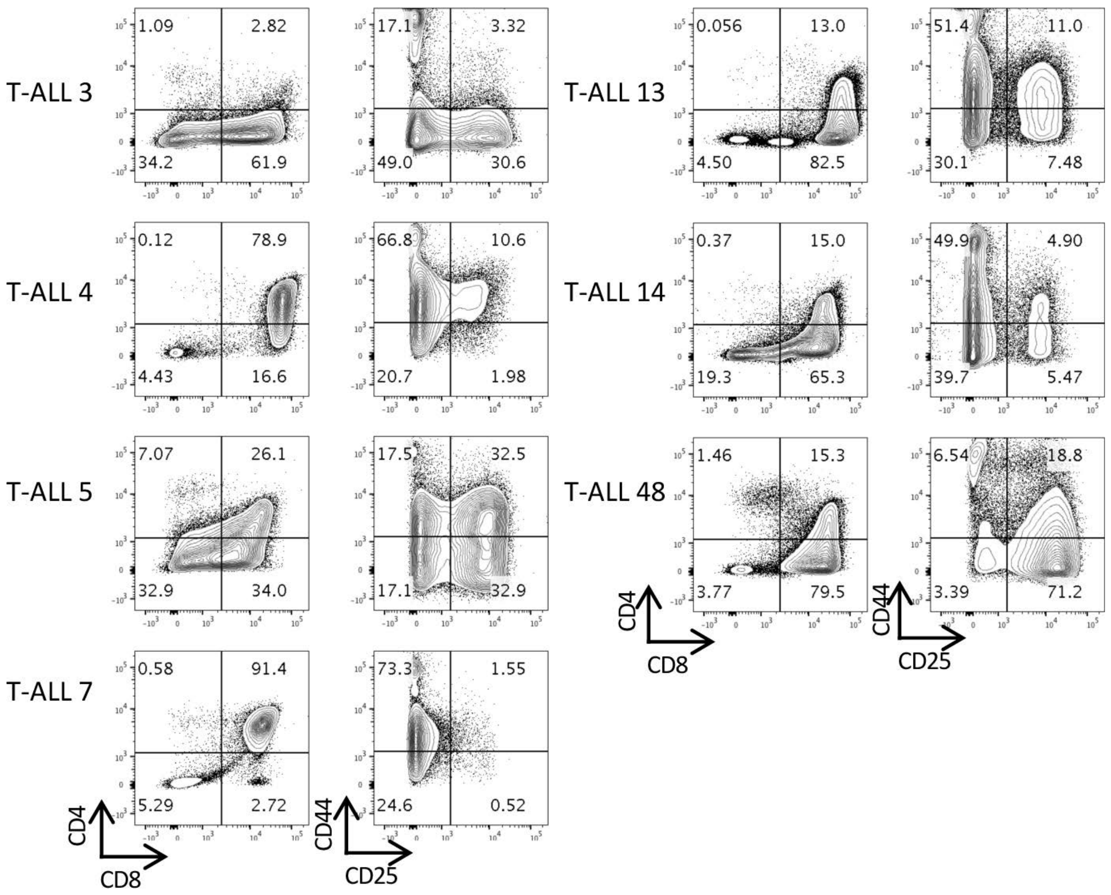

Fig. S2. Immunophenotype of individual T-ALL cases. Leukemic cells in spleen from different T-ALL bearing mice were analyzed for CD4, CD8, CD44 and CD25 expression by flow cytometry.

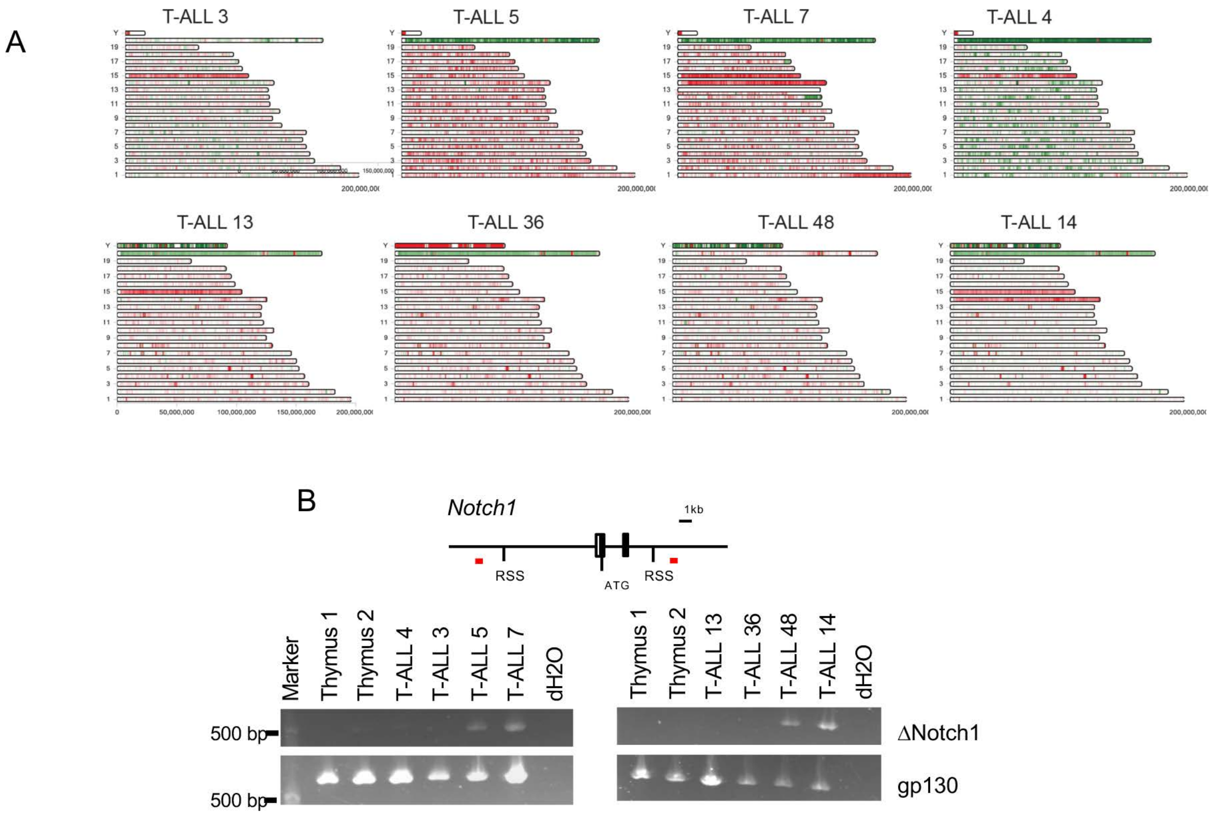

Fig. S3. Genomic characterization of individual murine T-ALL cancers. (A) Copy number variations at chromosome level in different T-ALL samples. Red represents gain and green represents loss of copy number. Chromosome 15 gain is due to frequent trisomy in mice. (B) Scheme of Notch1 genomic locus. RSS at position -8131 and +3573 are marked. Start codon is indicated, coding exons are in black. Positions of PCR primers used in analysis are marked in red. Genomic DNA from T-ALL and normal thymus was subjected to PCR analysis. The presence of a PCR product indicates 5' genomic deletion in the *Notch1* locus. The wild-type locus is too large to yield a PCR product. *gp130* was a positive loading control.

Disease Models & Mechanisms • Supplementary information

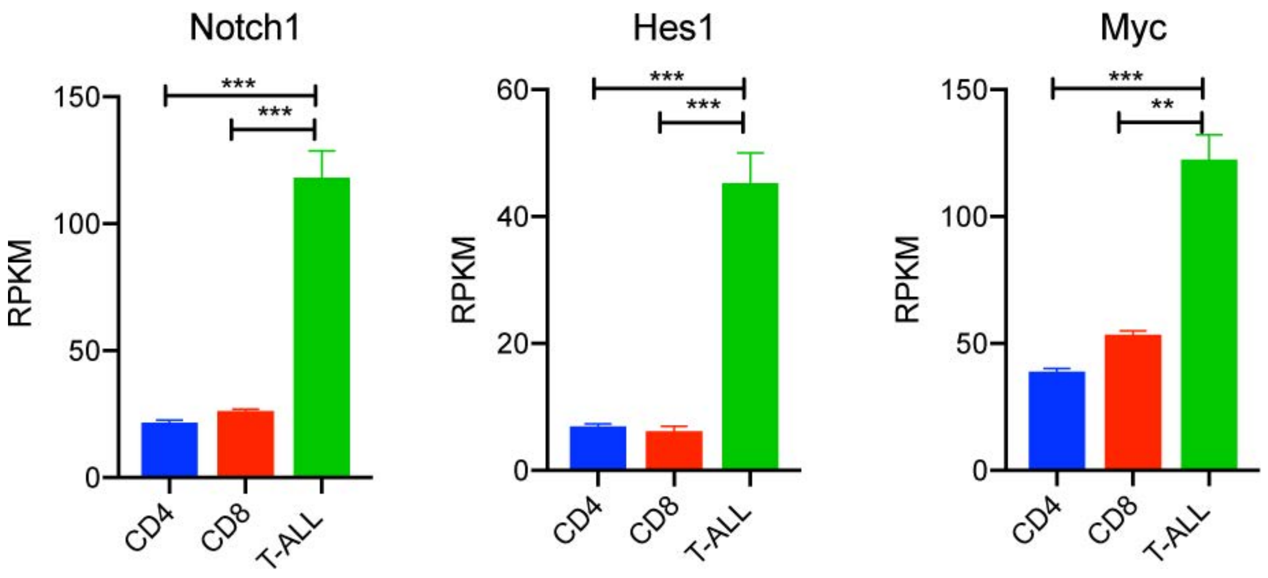

Fig. S4. Notch1 and its target genes were upregulated in T-ALL. Expression of indicated genes in spontaneous T-ALLs compared to normal T cells (CD4 or CD8) from C57Bl/6 spleen. Mean+/-SEM were plotted. *t*-test was performed between groups (****P*<0.001; *****P*<0.0001).

Disease Models & Mechanisms • Supplementary information

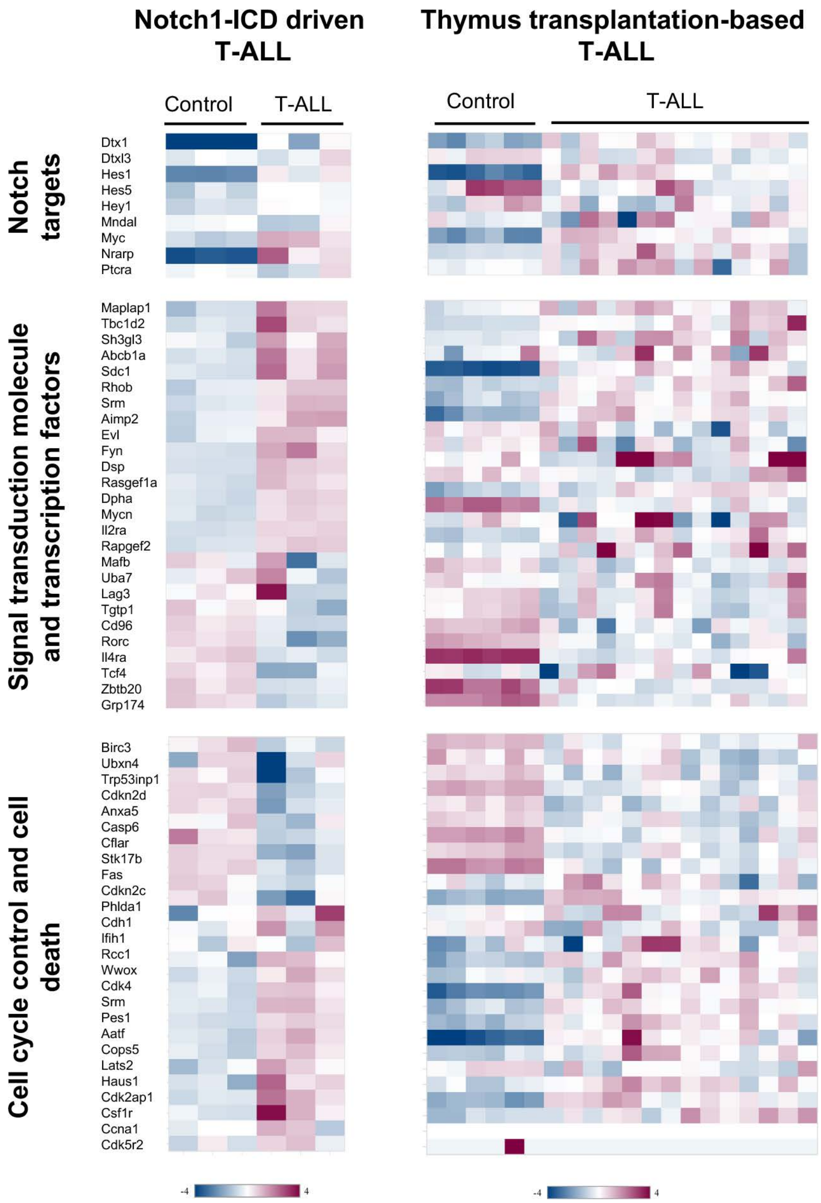

Fig. S5. Gene expression changes in Notch1-ICD driven and thymus transplantation-based T-ALL models. Heatmap of gene changes in Notch1-ICD T-ALL model (Li et al, J. Exp. Med. 2008) (left panel) compared to thymus transplantation-based T-ALL (right panel) relative to control. Each column represents one sample.

Disease Models & Mechanisms • Supplementary information

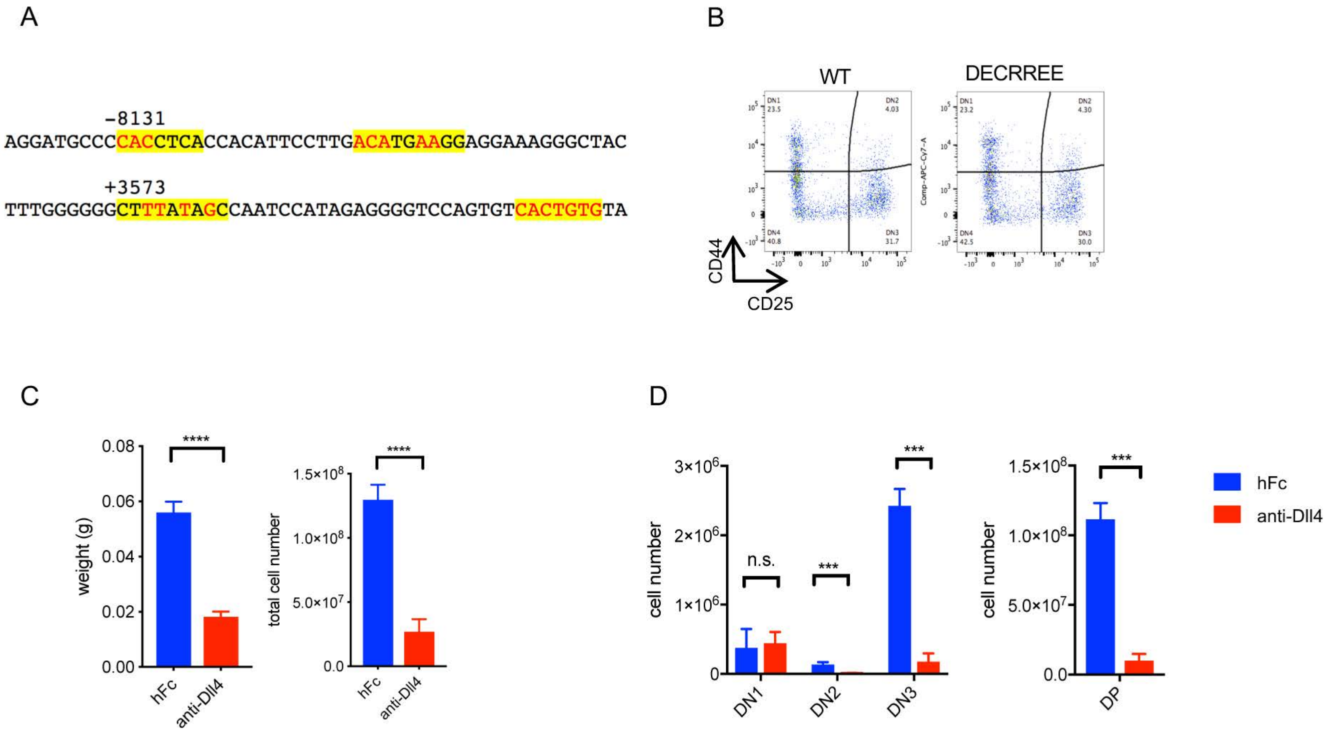

Fig. S6. Normal thymic T cell development in Notch1^{DECRREE} mice. (A) Cryptic RAG recombination signal sequences (RSS) in the 5’ murine Notch1 locus (highlighted in yellow). Residues that match RSS consensus sequences are in red. (B) FACS analysis of CD4$^-$CD8$^-$ T cell populations in 3-month old thymus from wild type and Notch1 DECRREE mice. (C) Notch1^{DECRREE} mice were treated with either control or anti-Dll4 antibody (5mg/kg, once weekly for two weeks). Thymus weight and cellularity are shown. (D) Analysis of T cell populations of 3-month old thymus from Notch1^{DECRREE} mice treated with control hFc or anti-Dll4. DN1, CD4$^-$CD8$^-$CD44$^+$CD25$^-$; DN2, CD4$^-$CD8$^-$CD44$^+$CD25$^+$; DN3, CD4$^-$CD8$^-$CD44$^-$CD25$^+$; DP, CD4$^+$CD8$^+$. Mean+/-SEM were plotted. *t*-test was performed between groups (****P*<0.001; *****P*<0.0001).

Disease Models & Mechanisms • Supplementary information

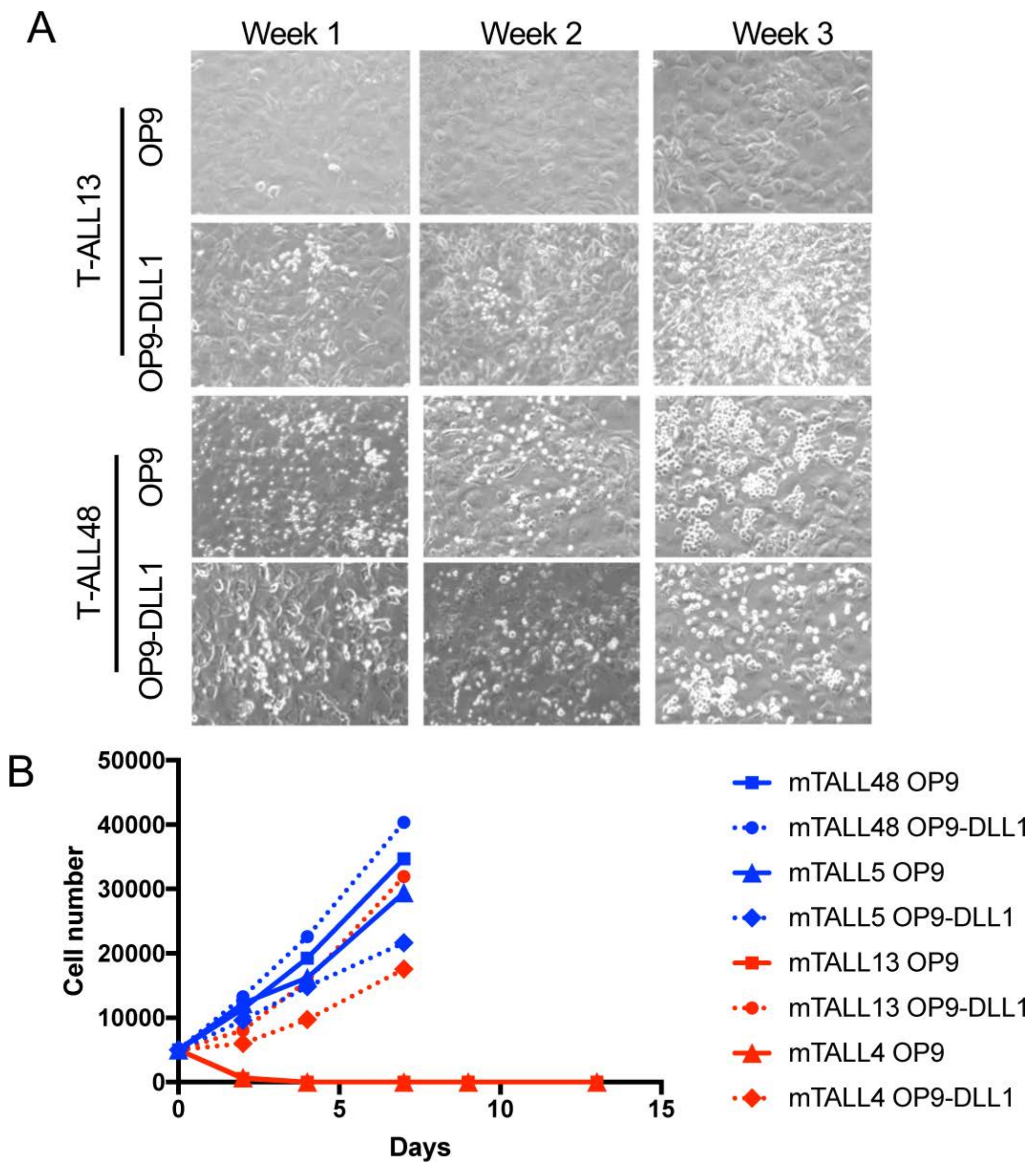

Fig. S7. *In vitro* growth of murine T-ALL different *Notch1* mutations. (A) Primary leukemic cells, isolated from spleen of mice with established T-ALL13 (Notch1 PEST domain mutation) and T-ALL48 (Notch1 5' deletion), were cultured in vitro on either OP9 stromal cells, or OP9 expressing the Notch ligand DLL1 (OP9-DLL1). Microscopic pictures were shown. (B) *In vitro* growth curves for T-ALL48 and T-ALL5, harboring Notch1 5' deletions and T-ALL13 and T-ALL4, characterized by Notch1 PEST domain mutations, on OP9 or OP9-DLL1 stromal cells..

Disease Models & Mechanisms • Supplementary information

A

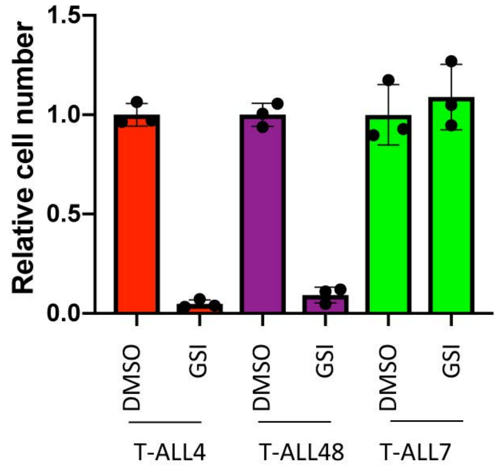

B

Sample ID	Notch1 somatic mutation domain	Notch1 5' deletion	Other genes mutated
T-ALL 4	PEST	-	
T-ALL 48	PEST	+	
T-ALL 7		+	Nras

Fig. S8. *In vitro* sensitivity of T-ALL cases to gamma secretase inhibitor (GSI). (A) A panel of representative primary T-ALLs were cultured on OP9-DLL1 feeder cells and treated with either DMSO or GSI (DAPT 10 uM for 96 hours). T-ALL4 cells with Notch1 PEST domain mutation and T-ALL48 cells with Notch1 PEST domain mutation and Notch1 5' deletion were sensitive to GSI treatment. T-ALL7 cells with Notch1 5' deletion and Nras mutation were resistant to GSI treatment. (B) Summary of Notch mutational status for T-ALL cases tested.

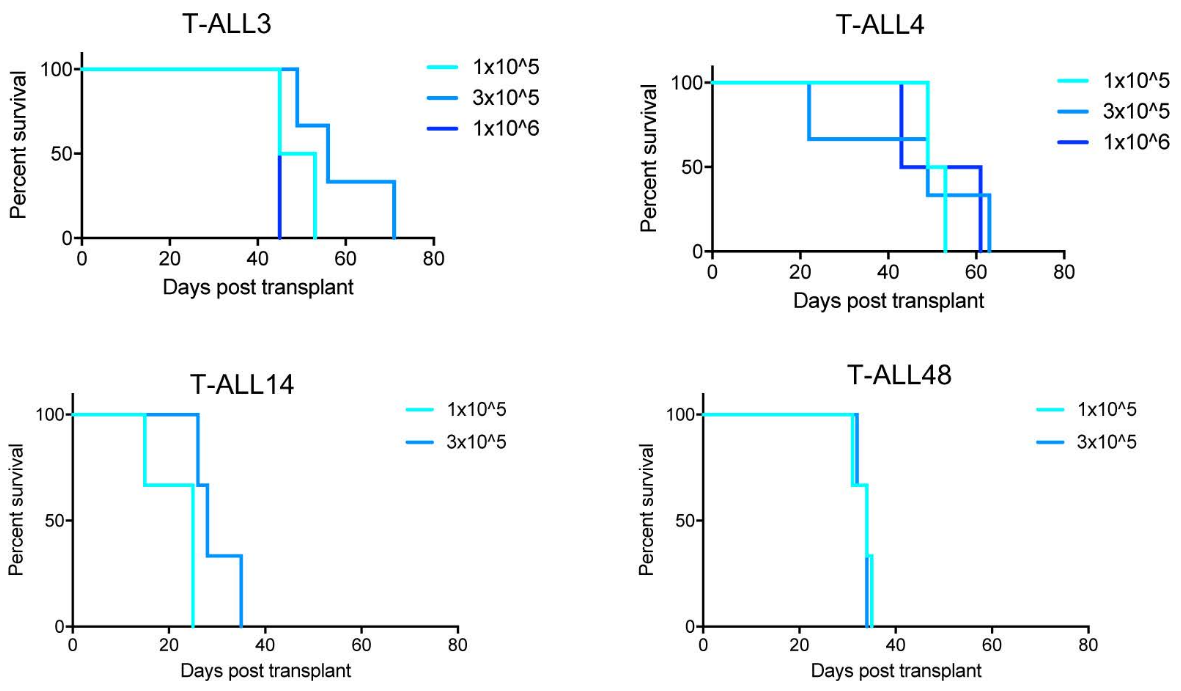

Fig. S9. Survival of immuno-deficient mice serially transplanted with primary murine T-ALL. Splenic leukemic cells form primary T-ALL3, 4, 14 and 48 were serially transplanted into *Rag2*$^{-/-}$*Il2rg*$^{-/-}$ (DKO) mice (*n*=3-4 for each group). Cell numbers transplanted are indicated.

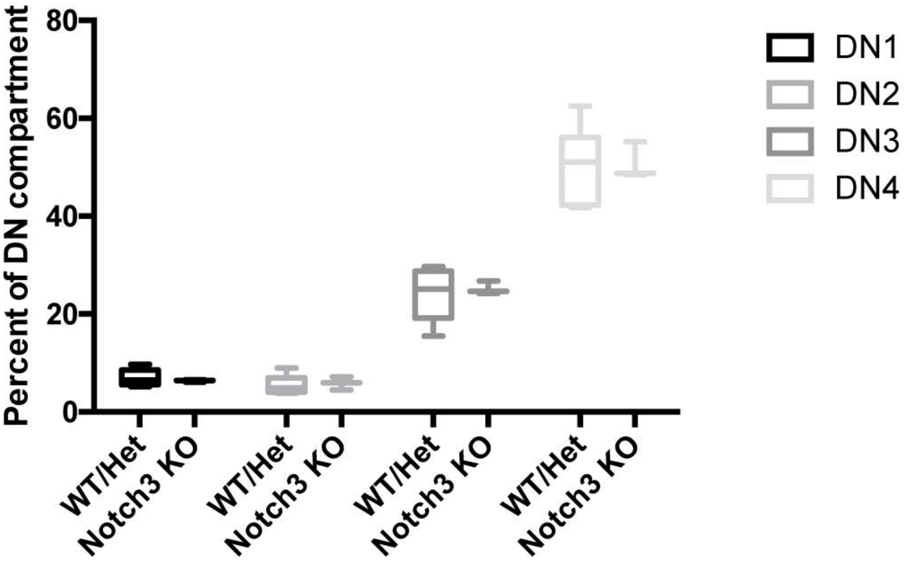

Fig. S10. Normal thymic T cell development in Notch3 deficient mice. Quantification of thymocytes in DN1-DN4 stages of development in WT and *Notch3* KO thymi. DN1-DN4 stages were defined as following: DN1: CD4$^-$CD8$^-$CD25$^+$CD44$^-$; DN2: CD4$^-$CD8$^-$CD25 $^+$CD44$^+$; DN3: CD4$^-$CD8$^-$CD25$^+$CD44$^-$; DN4: CD4$^-$CD8$^-$CD25$^-$CD44$^{-.}$ No statistically significant differences were observed.

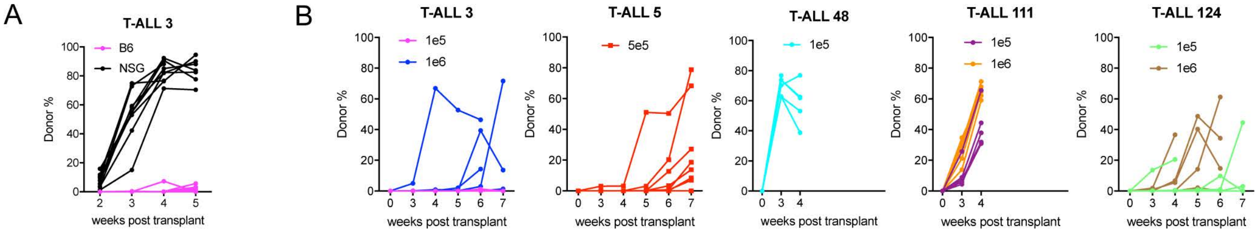

Fig. S11. Serial transplant of primary murine T-ALL into immunocompetent hosts. (A) $1x10^5$ T-ALL3 spleen cells were transplanted into immunocompetent C57Bl/6 (B6) mice or immunodeficient NSG mice. Donor-derived leukemic cells in peripheral blood were monitored over time. T-ALL growth was markedly delayed in C57Bl/6 mice relative to NSG mice. (B) Different numbers of leukemic cells ($1x10^5$ to $1x10^6$) from primary T-ALL cases were serially transplanted into immunocompetent C57Bl/6 mice. Donor-derived T-ALL cells in peripheral blood were monitored over time. Each line represents one mouse.

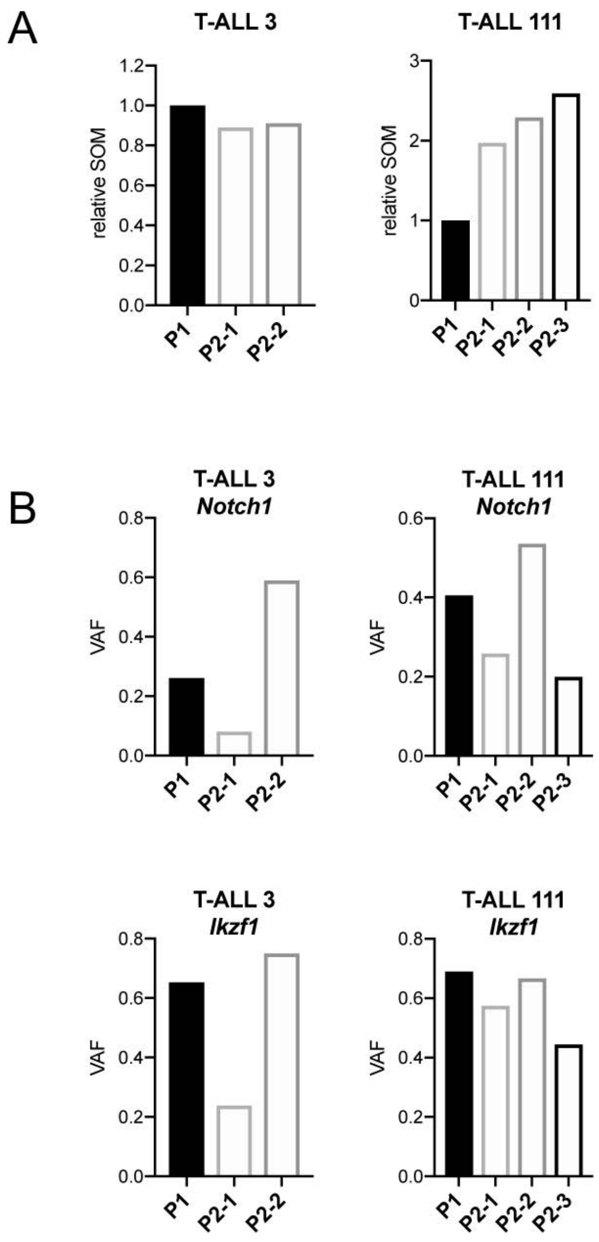

Fig. S12. Mutational burden in primary and secondary T-ALL in immunocompetent mice. (A) Splenocytes from secondary T-ALL 3 and T-ALL 111 were subjected to exome sequencing. Number of somatic mutations were compared between primary (P1) and secondary (P2) T-ALL. (B) Variant allele frequency (VAF) of Notch1 mutation and Ikzf1 mutation were compared between primary (P1) and secondary (P2) T-ALL. Each bar represents individual sample.

Disease Models & Mechanisms • Supplementary information

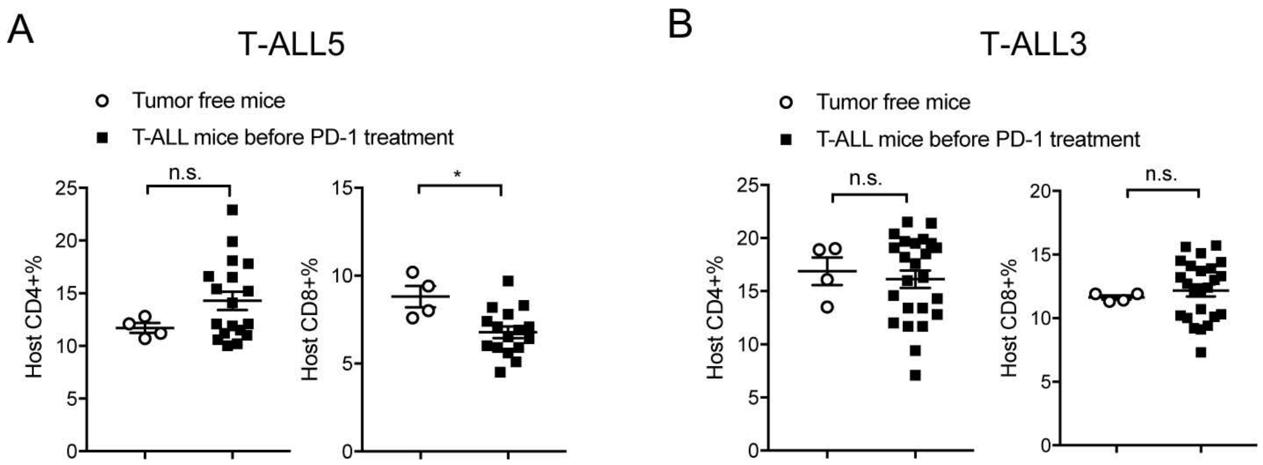

Fig. S13. Host T cell frequencies in C57Bl/6 mice at the start of anti-PD-1 treatment. 5x10^5 T-ALL5 (A) and T-ALL3 (B) leukemic spleen cells were transplanted into immunocompetent C57Bl/6 mice. Host T cell frequency (CD4$^+$% and CD8$^+$%) in peripheral blood were analyzed in T-ALL bearing mice at the start of anti-PD-1 treatment and in tumor free control mice. Each dot represents one mouse and bars represent mean+/-SEM. Treatment groups were compared by one-way ANOVA with Dunnett's multiple comparison test (*P<0.05; n.s. not statistically significant).

Disease Models & Mechanisms • Supplementary information

SUPPLEMENTAL METHODS

Exome sequencing data analysis. Read alignment and processing were performed using OSA aligner embedded in ArrayStudio. On average, around 80 million reads were generated for each sample and over 65% are mapped on targets, which gives 80% of targets with more 20x read coverage depth. Mutation (including small insertion and deletion) calling cutoffs used were: at least 10% variant allele frequency and at least 5 variant reads from both strands. Complete details of the pipeline can be found online at http://www.arrayserver.com/wiki/. We estimated somatic copy number variation patterns at the arm-level with whole exome sequencing (WES) data. Given DNA reads alignment input (BAM files) for a tumor sample and matched normal, the VarScan2 function embedded in ArrayStudio (www.omicsoft.com; www.arrayserver.com) was utilized to calculate read coverage depth ratio (log2Ratio) and summarize the copy number status. The average log2Ratio values for every 100kb sliding window per chromosome were displayed with Segment Chromosome View in ArrayStudio. One normal sample from DKO spleen served as control for all tumors generated on DKO strain background.

RNA sequencing and TCR analysis. RNA-Seq reads were mapped to the mouse mm10 transcriptome by OSA aligner (Hu et al., 2012) implemented in ArrayStudio software with 85% unique mapping rate on average. Quantification of gene expression and identification of fusion transcripts are also performed in ArrayStudio software. We then combined all unmapped reads and reads that mapped to TCR genes together to characterize TCR repertoire. These reads were analyzed by TCRklass (0.6.0) (Yang et al., 2015) with default option to get CDR3 profile and distribution. Here we used fraction of number of reads for top1 count compared to total clone reads to characterize the TCR clonality. If a sample has the fraction very close to 1, it has a dominant TCR clone in its TCR repertoire.

Hu, J., Ge, H., Newman, M., and Liu, K. (2012). OSA: a fast and accurate alignment tool for RNA-Seq. Bioinformatics *28*, 1933-1934.
Yang, X., Liu, D., Lv, N., Zhao, F., Liu, F., Zou, J., Chen, Y., Xiao, X., Wu, J., Liu, P., *et al.* (2015). TCRklass: a new K-string-based algorithm for human and mouse TCR repertoire characterization. J Immunol *194*, 446-454.

Disease Models & Mechanisms • Supplementary information
